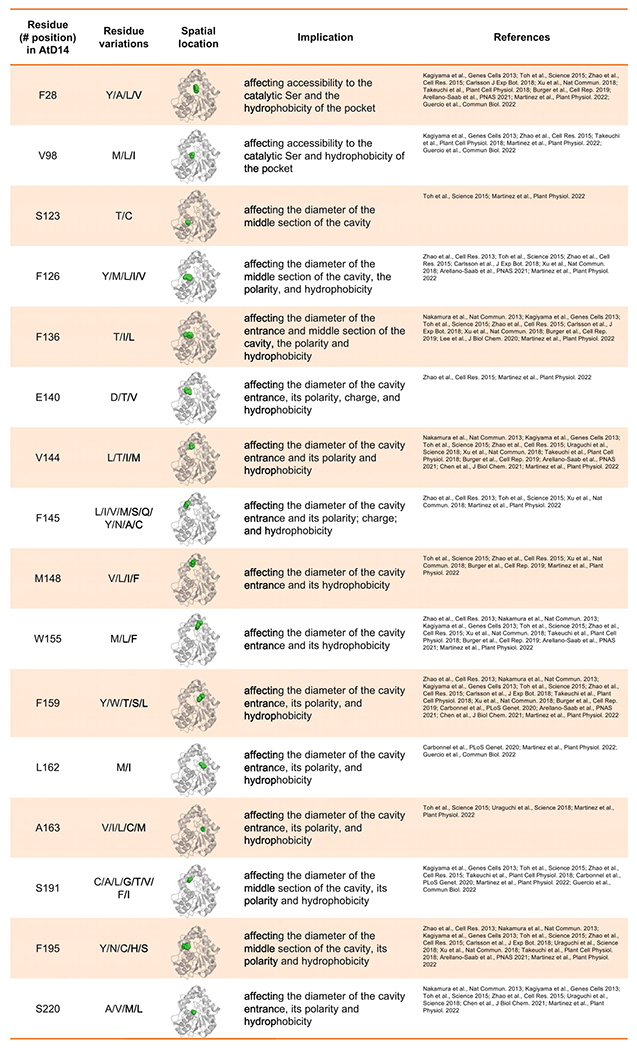# Strigolactones: diversity, perception, and hydrolysis

**DOI:** 10.1007/s11101-023-09853-4

**Published:** 2023-01-23

**Authors:** Angelica M. Guercio, Malathy Palayam, Nitzan Shabek

**Affiliations:** Department of Plant Biology, College of Biological Sciences, University of California – Davis, Davis, CA 95616, USA

**Keywords:** Strigolactone, Perception, Hydrolysis, Receptor, Phytohormone signaling

## Abstract

Strigolactones (SLs) are a unique and novel class of phytohormones that regulate numerous processes of growth and development in plants. Besides their endogenous functions as hormones, SLs are exuded by plant roots to stimulate critical interactions with symbiotic fungi but can also be exploited by parasitic plants to trigger their seed germination. In the past decade, since their discovery as phytohormones, rapid progress has been made in understanding the SL biosynthesis and signaling pathway. Of particular interest are the diversification of natural SLs and their exact mode of perception, selectivity, and hydrolysis by their dedicated receptors in plants. Here we provide an overview of the emerging field of SL perception with a focus on the diversity of canonical, non-canonical, and synthetic SL probes. Moreover, this review offers useful structural insights into SL perception, the precise molecular adaptations that define receptor-ligand specificities, and the mechanisms of SL hydrolysis and its attenuation by downstream signaling components.

## Introduction

Strigolactones (SLs) are a recently discovered class of phytohormones that have become the focus of numerous research studies in the last decade. SLs garner much attention because of their central role in modulating an increasingly wide range of plant-developmental and plant-environmental processes. Since their first discovery, SLs have been characterized to have remarkable dual function as both exogenously secreted signaling molecules and endogenous hormones.

The first identified SL was the strigol molecule, which was responsible for stimulating germination of *Striga* plants (Cook et al. 1966). Thereafter, an entire family of *Striga*-stimulating molecules were identified, and their unifying structural characteristic contains a lactone ring, hence their name (strigollactones) (Butler 1994). SLs when exuded by host plants’ roots can stimulate germination of nearby parasitic witchweeds of *Striga* and *Orobanche* species. As obligate parasites, members of *Striga* and *Orobanche* have little to no photosynthetic capability and depend entirely on the host organism for nutrients, assimilates, and water, posing a major threat to host plants and decimating crop yields (Parker 2009; Westwood et al. 2010). While SLs were discovered because of this role in parasitism, their exudation was also found to be of crucial function for the host plants. It was discovered that SLs serve as critical signals in establishing symbiotic relationships with arbuscular mycorrhizal fungi, that help the plants to take up nutrients from the soil (Akiyama et al. 2005).

As hormones, SLs were first identified to have endogenous roles in regulating shoot branching (Gomez-Roldan et al. 2008; Umehara et al. 2008). Later studies expanded the roles of SLs to modulate leaf growth, leaf senescence, secondary stem thickening, formation of adventitious roots, lateral roots, and root hairs. The list and networks of SL-dependent phenotypes continue to grow as their roles are studied in diverse species and contexts (Brewer et al. 2013; Ruyter-Spira et al. 2013; Bennett and Leyser 2014; Seto et al. 2014; Smith and Li 2014). Additional implications of SLs function as well as their crosstalk with other phytohormone signaling pathways such as auxin, cytokinin, abscisic acid, gibberellin, jasmonate, and salicylic acid, have been increasingly revealed in the recent years (Omoarelojie et al. 2019).

Due to this central role of SL signaling in plant development and plant-environment interactions, the research on the mode of SL perception has become a main focus with over 50 studies in 17 species at the genetic, phenotypic, biochemical, and structural level. Over the past decade, these studies have revealed a complex mode of perception and signaling in plants. A major leap in understating SLs perception is the identification of their receptor protein which also acts as an active catalytic enzyme (Hamiaux et al. 2012; Nakamura et al. 2013; Zhao et al. 2013). Since this discovery, the SL receptor has been described in several species and takes on the name of D14/DAD2/RMS3/HTL (DWARF14, DECREASED APICAL DOMINANCE2, RAMOSUS3, HYPOSENSITIVE TO LIGHT) (Arite et al. 2009; Hamiaux et al. 2012; Zhao et al. 2013; Toh et al. 2015; de Saint Germain et al. 2016). The SL receptor was found to be part of a larger family of proteins, many of which have diversified or co-evolved to sense specific butenolide compounds. This family is classified as the D14/KAI2 (KARRIKIN INSENSITIVE 2) family of receptors (Bythell-Douglas et al. 2017). In order to better understand the mechanism of signal perception and transduction by D14 family receptors, several synthetic SLs including agonists, antagonists, and other experimental probes have been generated as research tools to further explore the complexity of SL perception mechanisms and the potential applications of these synthetic molecules for research and agriculture. Here we review SL perception including the diversity of SL molecules and the recent advancements in understanding their selectivity and hydrolysis mechanisms alongside the divergence between receptor proteins.

## Strigolactones as phytohormones

### Structural diversity of strigolactones

**Fig. 1:**
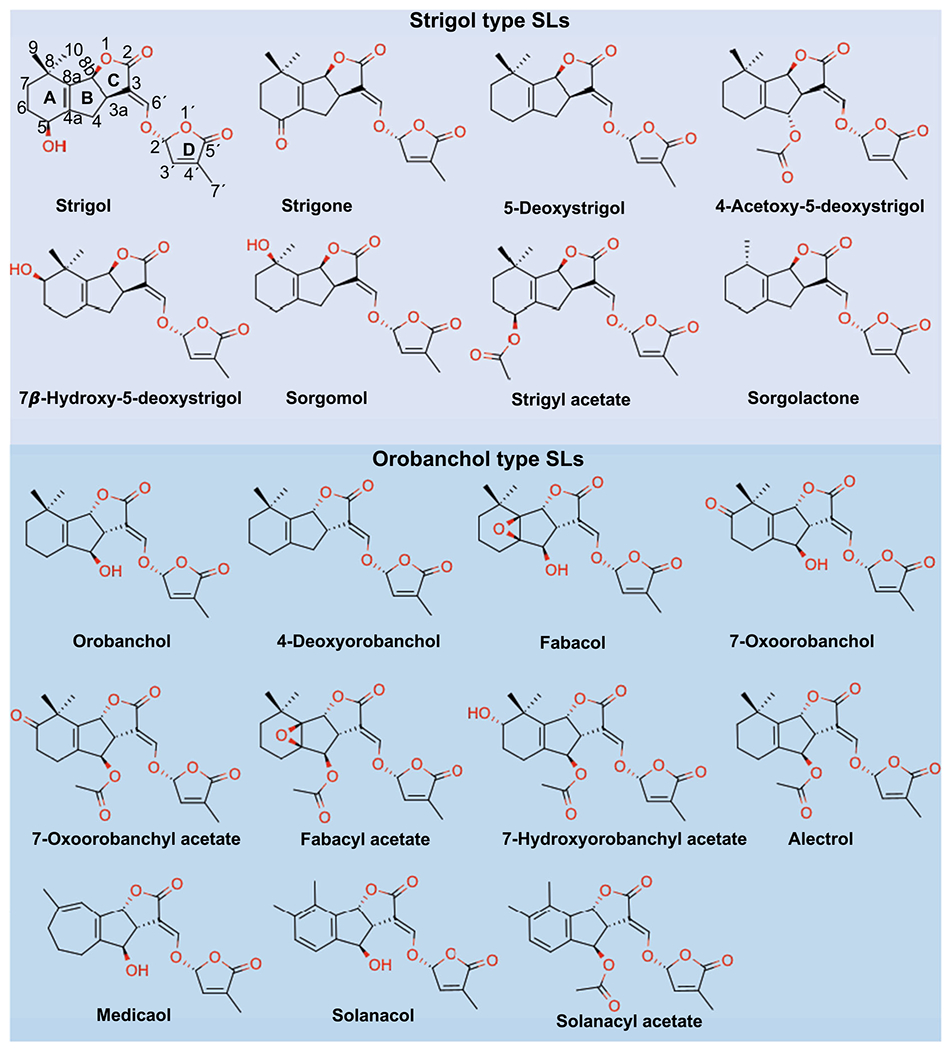
Structure and classification of canonical strigolactones. Strigol type SLs are shown in the upper panel (light purple shade background, in β-orientation at B-C ring junction). Orobanchol type SLs are shown in the lower panel (light blue shade background, in α-orientation at B-C ring junction). The conserved D-ring is shown in 2′R configuration for all structures

Plants produce mixtures of structurally diverse SLs. This endogenous array can vary between as well as within plant species. The first natural SL was isolated from the root exudates of cotton and identified to be the germination stimulant of parasitic *Striga*, taking on the name strigol (Cook et al. 1966). Since then, many more compounds such as sorgolactone, alectrol, and orobanchol have been identified from the root exudates of diverse plant species (Fig. 1) (Hauck et al. 1992; Müller et al. 1992; Yokota et al. 1998; Mori et al. 1999; Delaux et al. 2012). This group of carotenoid-derived terpenoid lactones molecules are collectively named strigolactones. Strigolactones are either classified as canonical or non-canonical based on their chemical structure. Here we discuss the discovery of a diversity of canonical and non-canonical SLs as well as the novel synthetic probes that have been generated to better study this emerging field.

### Canonical strigolactones

Naturally occurring canonical strigolactones contain the characteristic feature of a tricyclic lactone ring (ABC scaffold) which is connected to a common butenolide ring (D-ring) through the conserved 2′R configured enol-ether linkage (Butler 1994; Zwanenburg et al. 2009; Zwanenburg and Pospíšil 2013; Zwanenburg et al. 2016a, b; Yoneyama et al. 2018) (Fig. 1).

The ongoing identifications of diverse SLs reveal a variability in the C-ring configuration and provide further classification of SLs that are originally derived from 5-Deoxystrigol (5DS) with β-oriented C-ring as strigol-type (Motonami et al. 2013), and SLs that are derived from 4-Deoxyorobanchol (4DO) with an α oriented C-ring as orobanchol-type subfamilies (Zhang et al. 2014). In addition to the differences in the C-ring configuration, the AB rings in all canonical SLs can be derivatized through hydroxylation, methylation, acetylation, ketolation, and epoxidation (Al-Babili and Bouwmeester 2015). The substitution of the A-ring to benzene also diversifies the growing list of SLs from plants (Xie et al. 2007). To date, nearly 30 distinct naturally occurring SLs have been identified (Fig. 1), with diverse roles in growth, development, and as plant-environment signaling molecules. While some plant species such as tomato, petunia, pea, and poplar synthesize only orobanchol-type SLs, tobacco and sorghum can produce both types of canonical (orobanchol and strigol types) SLs (Xie et al. 2007; Mohemed et al. 2016; Gobena et al. 2017).

The canonical SLs diversity and species-specificity has been shown to be largely controlled by a central biosynthesis cytochrome P450 enzyme, CYP722C. CYP722C is suggested to be the key player in synthesizing both strigol and orobanchol type SLs from the SL precursor molecule carlactone (Wakabayashi et al. 2019, 2020; Mori et al. 2020a, b). For example, in *Solanum lycopersicum (Sl)*, SlCYP722C was found to be necessary for the synthesis of orobanchol-type SLs (Wakabayashi et al. 2019). Similarly, CYP722C from *Lotus japonicus* and *Gossypium arboreum* were implicated in the biosynthesis of strigol-type SLs (Mori et al. 2020a, b; Wakabayashi et al. 2020). For more comprehensive review of SLs biosynthesis, we recommend several excellent reviews including Yoneyama and Brewer and Zorrilla et al. (Yoneyama and Brewer 2021; Zorrilla et al. 2022). Future investigations of CYP722C genes from various plants will shed light on the biochemical diversification of canonical SLs and how this large repertoire arose in different plant species.

### Non-canonical strigolactones

**Fig. 2:**
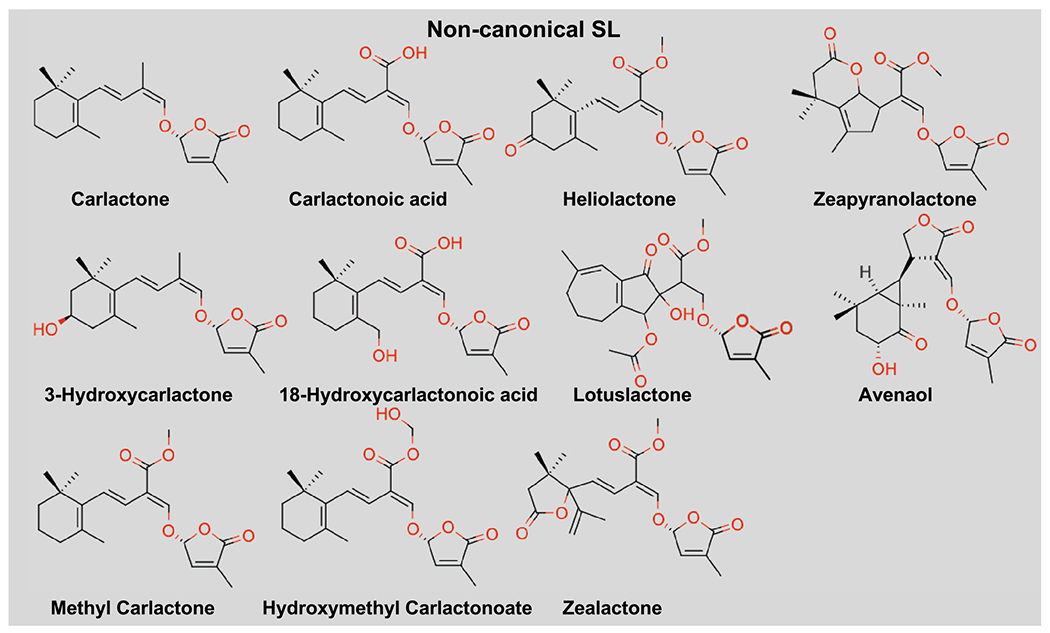
Structure of non-canonical strigolactones. Non-canonical strigolactones retains the intact lactone D-ring in 2′R configuration that is connected to distinct moieties

Non-canonical SLs are characterized as molecules that lack the typical ABC-ring yet contain the 2′R configured enol-ether linkage and D-ring moiety (Kim et al. 2014; Umehara et al. 2014; Charnikhova et al. 2017; Xie et al. 2017, 2019) (Fig. 2). Non-canonical SLs represent any SL that does not fall into the canonical category. While there are no defined classes for non-canonical SLs nor a common core enzyme or precursor, the biosynthesis enzymes in different species are responsible for the resulting diversity of the molecules. For example, Lateral Branching Oxidoreductase (LBO) in *Arabidopsis* and its homologs in maize, sorghum and tomato, are involved in the metabolism of the non-canonical SL precursor molecule Methyl-carlactonate (MeCLA, a derivative of carlactone) (Yoneyama et al. 2020). The LBO enzyme can convert MeCLA to hydroxymethylCLA, both of which seem to be bioactive non-canonical SLs (Alder et al. 2012; Abe et al. 2014; Seto et al 2014; Yoneyama et al. 2018; Mashiguchi et al. 2022). Additionally, MeCLA can be further derivatized and its substructures have been found in various species such as helicolactone from sunflower, lotuslactone from lotus and methyl zealactone from maize (Ueno et al. 2014; Charnikhova et al. 2017; Xie et al. 2017, 2019). One identified enzyme that is involved in this derivatization is 2-oxoglutarate-dependent dioxygenase, which was found to play role in the biosynthesis of lotuslactone (Mori et al. 2020a, b).

Interestingly, some plant species such as *Arabidopsis*, maize, and poplar produce both canonical and non-canonical SLs. Therefore, further identification of new metabolic precursors and SLs biosynthesis enzymes will illuminate the diversification of canonical and non-canonical SLs across plants.

### Synthetic strigolactones as research tools

Both canonical and non-canonical SLs are generally unstable compounds that can spontaneously disintegrate into inactive ABC and D-ring products in alkaline conditions (Yoneyama and Brewer 2021). Additionally, the laborious process to isolate the natural SLs from plants yields only trace amounts (picomolar to nanomolar) (Yoneyama and Brewer 2021). Therefore, a multitude of synthesis efforts have been made including the first reported synthesis of strigol as early as 1966 (Cook et al. 1966). Since this breakthrough, several procedures are now available to synthesize various derivatives of SLs. Among the methods, the most common way to synthesize SLs starts with the preparation of ABC scaffold followed by selective oxidation of either A-ring or B-ring to produce strigol or orobanchol type precursors. The synthesis is then completed by the addition of a butenolide ring connected via an enol-ether linkage, which yields the racemic mixtures of strigol/orobanchol and their corresponding enantiomers (2′epimers) (Zwanenburg and Pospíšil 2013; Zwanenburg et al. 2016a, b). These mixtures are often purified through enantioselective High Performance Liquid Chromatography (HPLC) or through the asymmetric synthesis to yield pure and distinct SL enantiomers. While this results in higher yield than isolating SLs from plants, synthetic preparation of natural SLs are often time consuming, not scalable and generate poor yields regardless of the methods (either racemic or pure enantiomer) (Zwanenburg et al. 2016a, b).

**Fig. 3:**
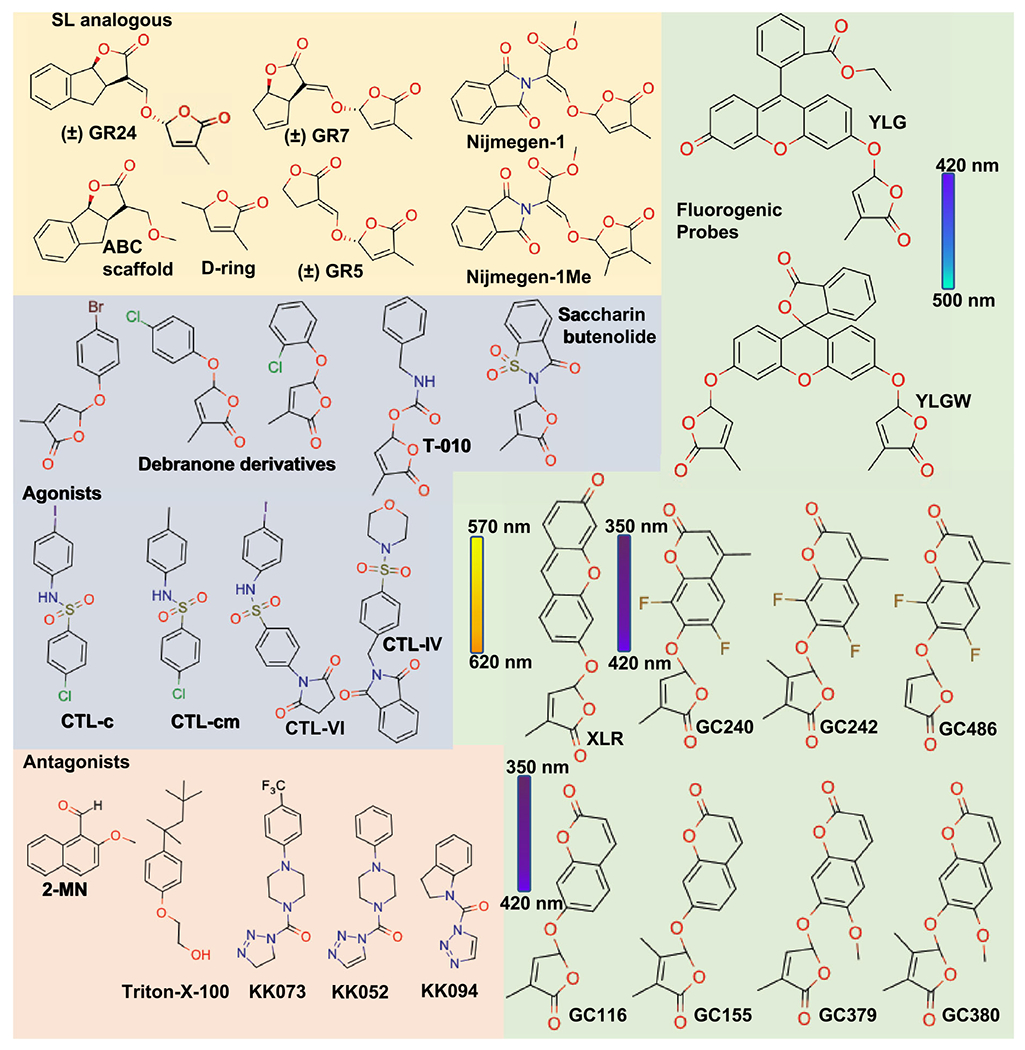
Structures of representative strigolactone analogs, agonists, antagonists, and fluorogenic probes. Strigolactone (SL) analogs contain intact D-ring that are connected to distinct ring system via enol-ether bridge, shown in upper left panel (pale yellow background). SL agonists/mimics retains the intact D-ring but lacks the enol-ether linkage, shown in middle-left panel (light purple background). SL antagonists lacking both the conserved enol-ether bridge and the intact D-ring, shown in lower left panel (pink background). In the structures of fluorogenic probes shown in right panel (pale green background), the ABC tricyclic lactone ring is replaced by various fluorescent moieties that are connected to the D-ring. In all the relevant structures, the D-ring is shown in 2′R configuration

The great efforts to generate simplified versions of SLs enabled the development of the most widely used SL analogs (or agonists), the GR compounds such as GR24, GR7 and GR5 (Fig. 3), named after Gerald Rosebery (Johnson et al. 1976). Similar to the synthesis of natural SLs, the production of GR24 initiates with the preparation of ABC scaffold from 1-indanone which is then attached to the chiral butenolide ring and thus generates two diastereoisomers and their corresponding enantiomers possibly mimicking the deoxy SLs (5DS and 4DO) (Mangnus et al. 1992). The GR24 with the configuration mimicking the 4DO is generally omitted as it was reported to be less active in germinating the parasitic plants whereas the GR24^5DS^ and GR24^ent–5DS^ are retained as a racemic mixture ( ±)-GR24 and referred as *rac*-GR24 or maintained as pure enantiomers. Molecules lacking the A-ring and AB-ring of GR24 such as GR7 and GR5 were also shown to be bioactive *Striga* germination stimulants (Nefkens et al. 1997). *rac*-GR24 is the most widely used compound to study the inhibition of shoot branching, the activity of SL receptor, and as a stimulant for *Striga* management (Johnson et al. 1976; Mangnus and Zwanenburg 1992). However, compounds with A-ring, AB-ring, D-ring, D-ring with ethoxy group, methyl substituent of D-ring, and molecules lacking the D-ring were shown to be biologically inactive in inducing *Striga* germination (Mangnus and Zwanenburg 1992; Zwanenburg et al. 2009; Zwanenburg et al. 2016a, b) or downstream signaling (Hamiaux et al. 2012). Therefore, the bioactiphore of strigolactone lies in the D-ring and is essential for bioactivity (Mangnus and Zwanenburg 1992; Zwanenburg et al. 2009; Zwanenburg et al. 2016a, b). This inspired the synthesis of various new SL mimic molecules that lack the ABC scaffold but retain the D-ring structure (Fukui et al. 2011, 2013; Boyer et al. 2014; Takahashi et al. 2016). This includes saccharine (Zwanenburg and Mwakaboko 2011), furanone derivatives like debranone, carbamate derivative T010, and phthalimide derivatives such as nijmegen-1, nijmegen-1Me (Fig. 3) (Nefkens et al. 1997; Samejima et al. 2016).

The development of SL agonists also led to the synthesis of fluorogenic probes such as Yoshimulactone green (YLG) (Tsuchiya et al. 2015), Xilatone Red (XLR) (Wang et al. 2021), and Guillaume Clave series (GC) compounds (de Saint Germain et al. 2022) (Fig. 3). These were designed as tools to investigate and understand the mechanism of SL perception and monitor its hydrolysis. Generally, these probes are designed as molecules comprise of the butenolide D-ring attached to the various editable fluorophores. The fluorescent signals of these pro-fluorophores are detected only after hydrolysis. This enables monitoring of the enzymatic activity and serves as a signal reporter of the SL receptor in vitro (Tsuchiya et al. 2015; de Saint Germain et al. 2016; Wang et al. 2021) and *in planta* (Tsuchiya et al. 2015; Wang et al. 2021).

Interestingly, a group of sulfonamide-related compounds called cotylimides (CTLs) which lacks the D-ring has been identified (Tsuchiya et al. 2010) (Fig. 3). These molecules can bind AtHTL/KAI2 and mediate the interaction with downstream signaling component MAX2 (see section below) yet have not been shown to serve as hydrolytic ligand by these receptors (Toh et al. 2014). Such molecules have the potential to be utilized as probes to study the distinct function and biological consequences of non-hydrolysable SLs.

In addition to SL agonists, various antagonists were also discovered through virtual screening of compounds that could fit into the catalytic cavity of the SL receptors (Mashita et al. 2016). Among the tested compounds is the 2-Methoxy-1-Naphthaldehyde (2-MN) that has been shown to inhibit the interaction of D14 with D53 (downstream SL signaling protein, see section below), rescue the rice tillering buds suppressed by SL, and has an inhibitory effect on SL-induced germination of *Striga*. Another antagonist molecule, soporidine, was identified in a chemical screen and was shown to bind *Striga* HTL (ShHTL) and inhibit *Striga* germination (Holbrook-Smith et al. 2016) (Fig. 3). Moreover, the detergent, Triton-X-100, was found in a crystal structure of ShHTL7 and proposed to block the catalytic pocket resulting in a moderate inhibitory impact on *Striga* germination (Sahul Hameed et al. 2022). Recently, a more potent antagonist piperazine derivative, dormirazine, has been identified and shown to occupy the catalytic cavity of ShHTL7 and inhibit *Striga* germination (Arellano-Saab et al. 2022). Lastly, triazole urea derivatives named KK compounds, were developed to serve as covalent inhibitors by binding the catalytic serine of rice D14. Among the KK derivatives that were reported to impact SL signaling either as agonists or antagonists, KK094 was found to be the most potent antagonist exhibiting SL signaling inhibition in rice (Nakamura et al. 2019; Jamil et al. 2021) (Fig. 3). Together, the continuous efforts to develop SL agonist and antagonists further underline the increasing demand to synthesize better molecular probes to serve in agricultural applications as well as research tools to study SL perception and signal transduction.

## Strigolactone perception by D14 family proteins

### Identification of strigolactone receptors

The receptor for SL was first identified as a dwarf mutant in rice that was later characterized as SL-insensitive, named DWARF14 or OsD14 (*Oryza sativa*) (Ishikawa et al. 2005; Arite et al. 2009). This was followed by the identification and characterization of these proteins as definitive SL receptors in petunia, DAD2 (Hamiaux et al. 2012). Then, the D14 ortholog in *Arabidopsis thaliana*, AtD14 was identified as well as the paralogous D14 family receptor KAI2 (Waters et al. 2012). Following these findings, an increasing number of studies identified and examined of SL-receptor function in many other plant species including chrysanthemum (Wen et al. 2015), *Medicago* (Lauressergues et al. 2015), barley (Marzec et al. 2016), poplar (Zheng et al. 2016), pea (de Saint Germain et al. 2016), soybean (Ahmad et al. 2020), cotton (Wang et al. 2019), lotus (Carbonnel et al. 2020), wheat (Liu et al. 2021), canola (Stanic et al. 2021), sugarcane (Hu et al. 2021), tobacco (Li et al. 2020; White et al. 2022), and importantly in SL-induced parasitic plants such as *Striga* and *Phelipanche ramosa* (Toh et al. 2015; de Saint Germain et al. 2021). Additionally, sequence analyses identified putative SL receptor homologs in 143 species and classified evolutionary sub-families within the larger α/β D14 family receptors including D14s and related butenolide receptors–KAI2s as well as DLK2s (D14-LIKE2) (Bythell-Douglas et al. 2017). The characterization of SL-receptors in these and many other plant species as well as their spatio-temporal expression are subjects of ongoing investigation in this field.

### The specificity of strigolactone receptors

D14 receptors are able to perceive a wide breadth of SLs, and their distinct diversification within the family and between species have allowed for extensive ligand sensitivities. For example, the extended D14/KAI2 family of enzymes exhibit mutually exclusive functions in plants even though D14 and KAI2 are evolutionarily related. D14 and KAI2 have specific ligand selectivity for different stereoisomers of butenolide compounds. In general, D14 receptors preferentially perceive SLs where the D ring is in 2′R configuration (Fig. 1), whilst KAI2 receptors prefer the 2′S configuration (Scaffidi et al. 2014; de Saint Germain et al. 2016; Carbonnel et al. 2020; de Saint Germain et al. 2021; Guercio et al. 2022). Even within D14 and KAI2 families, these receptors have acquired adaptive sensitivity to different species and/or context-specific SLs, allowing these receptors to represent a wider diversity of ligand specificities.

### α/β hydrolase fold

**Fig. 4:**
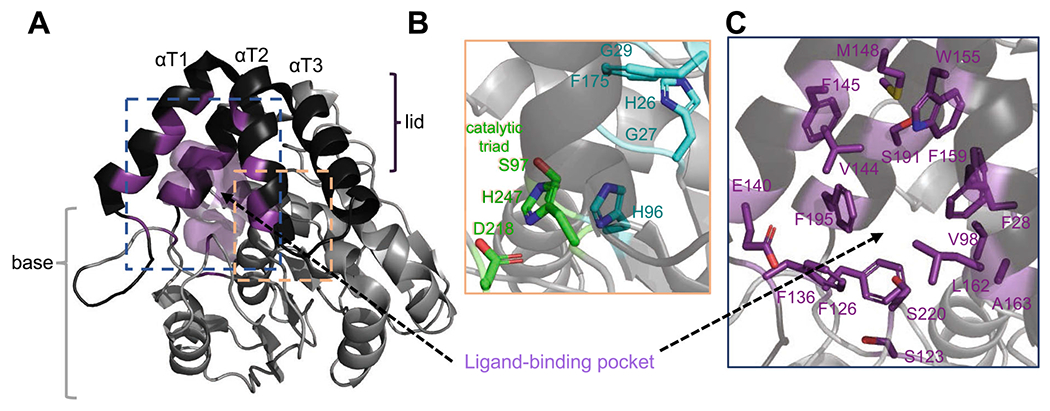
Overview of the structure of strigolactone receptor and its SL-binding pocket. **A** Representative *Arabidopsis thaliana* D14 structure (based on PBD ID: 4IH4) is shown as cartoon. **B** Close-up view into the SL-binding pocket. Invariant residues are shown in cyan sticks and the catalytic triad in green sticks. **C** Diverged residues (see also Table 1) are shown in purple sticks. 3D structure illustration and analysis were generated using PyMOL Molecular Graphics System, Schrödinger, LLC

α/β hydrolases represent a large family of enzymes present in all living organisms. In plants, α/β hydrolases are implicated in several cellular processes including signal transduction pathways (phytohormone SLs, GAs, and karrikin/KAI2-ligand) (Shimada et al. 2008; Hamiaux et al. 2012; Kagiyama et al. 2013; Mindrebo et al. 2016). First described by Ollis et al. in 1992, the α/β hydrolase fold is comprised of a core 8-stranded β-sheet surrounded by α-helices (Ollis et al. 1992). As members of the α/β hydrolase superfamily, D14s have a subset of ~ 4 helices that form a lid whilst the remaining helices and beta strands form a base (Fig. 4A). This assembly forms a largely hydrophobic ligand-binding pocket centered in between the lid and the base (Kagiyama et al. 2013). Typical to serine hydrolases, the serine catalytic triad is structurally positioned in the rear of the ligand-binding pocket and considered to be functionally active for all D14s and KAI2s (Fig. 4B). This is not the case for the GA receptor protein, GID1, where the catalytic histidine has been substituted to valine. GID1, therefore, acts solely as a receptor and not as an enzyme (Shimada et al. 2008; Mindrebo et al. 2016). Therefore, the dual receptor-hydrolase function of D14s/KAI2s represents a unique mode of hormone perception in phytohormone signaling pathways (Mindrebo et al. 2016).

### Strigolactone binding pocket: from structure to function

The advancements in resolving the crystal structures of D14 and KAI2 receptors have enabled a deeper understanding of perception mechanisms and revealed structurally similar receptors with distinct functions (Table 1). In the past decade an increasing number of studies have investigated the causal divergences that result in differential ligand selectivity between D14 orthologs and paralogs. The hydrophobic ligand-binding pocket has been a topic of great interest because of its importance in SLs accessibility and perception. Across species and paralogs, the D14 structures provide evidence on how sequence variation can alter the receptor towards different ligands. Analysis of the SL-binding pocket morphology of D14s shows alterations in pocket entrance, diameter, shape, as well as the pocket depth, width, and accessibility to the catalytic serine (Table 1 and Fig. 4B). For example, the *Striga* SL receptor, ShHTL7, has been proposed as a hyper-sensitive receptor *in planta* (picomolar sensitivities for 5DS and sorgolactones, and nanomolar sensitivities to strigol (Toh et al. 2015)). ShHTL7 can also perceive a larger compilation of SL molecules, and its pocket is amongst the largest in size, diameter, and volume. On the other hand, ShHTL1 seems to be much less perceptive to synthetic SLs, likely due to a much smaller binding pocket (Table 1) (Toh et al. 2015; Xu et al. 2018).

These differences in ligand sensitivity and pocket size have been correlated to specific amino acid alterations that fall into two broad classes. One class includes mutations in the conserved connecting loops that participate in positioning the helices of the receptor’s lid, which directly affect the pocket size, shape, and accessibility (Xu et al. 2018; Bürger et al. 2019; Bürger and Chory 2020; Lee et al. 2020) (Fig. 4A). Examples include divergence within *Striga* HTLs which contain either a tyrosine or phenylalanine at position 150 (Y152 in *Arabidopsis*) on the loop connecting αT1–αT2 helices (Fig. 4A) (Xu et al. 2018). A substitution in HTLs 4, 5, and 7 to phenylalanine at this residue results in loss of hydrogen bonds between helices αT1 and αT3, which increases the plasticity and creates a larger, more accessible pocket, and thus a more ligand-sensitive receptor (Fig. 4A and Table 1) (Xu et al. 2018; Bürger and Chory 2020). Similarly, in *Physcomitrella patens* (Pp), the ancestral D14 family proteins have an altered pocket size, due to a S166A substitution in the loop connecting αT2-αT3 (Bürger et al. 2019) (166 in *Arabidopsis* Fig. 4A and Table 1). Here again, the serine to alanine substitution results in the loss of a hydrogen bond which enlarges the binding pocket and increases ligand sensitivity for all PpKAI2-like proteins containing this substitution (Bürger et al. 2019; Bürger and Chory 2020). The second class of substitutions informing divergence in ligand selectivity includes mutations that directly alter the SL-binding cavity, which is the focus of the next section

### Diverged pocket residues inform differential ligand selectivity

The SL binding pocket, while only composed of ~ 25 amino acids (12% of total protein length), can harbor substitutions that alter ligand selectivity between paralogs or orthologs (Table 2). Among these residues, eight are invariant (positions relative to AtD14: H26, G27, G29, H96, S97, F175, D218, H247) including the three catalytic triad residues S97, D218, and H247 (Bythell-Douglas et al. 2017) (Fig. 4B). The conservation of these residues across the D14/KAI2 family is likely to maintain the receptors’ function and ligand accessibility. This is exemplified wherein the mutation of G28D in pea resulted in drastic overall instability of RMS3 (position relative to AtD14 = G27D, Fig. 4B) (de Saint Germain et al. 2016).

Sixteen pocket residues have been shown in multiple studies to evolutionarily diverge between and/or within species and as a result alter ligand selectivity. These 16 residues relative to AtD14 are in positions 28, 98, 123, 126, 136, 140, 144, 145, 148, 155, 159, 162, 163, 191, 195, and 220, and are highlighted in Table 2 and Fig. 4C. Orthologs have evolved differential ligand selectivity likely to perceive taxon-specific signaling molecules (Mindrebo et al. 2016), while paralogs exhibit differential selectivity likely to allow a specie to perceive a diversity of SL molecules (Carbonnel et al. 2020; Guercio et al. 2022). Recent studies examined the ability to direct ligand specificity by swapping residues between diverged D14 family paralogs (Tables 1 and 2) (Carbonnel et al. 2020; Arellano-Saab et al. 2021; Guercio et al. 2022). For example, substitutions in residues L160M and S190L (AtD14: L162M and S191L) are necessary to alter ligand selectivity between paralogous receptors in pea (Guercio et al. 2022). Similarly, in lotus, divergence between paralogs at the same positions as well as F157W (residue 159 in AtD14) were sufficient to swap selectivity (Carbonnel et al. 2020). Another study in *Arabidopsis*, shows that swapping three endogenous residues W153L, F157T, and G190T (positions relative to AtD14: 155, 159, 191), are sufficient to switch the karrikin (KAR) receptor, KAI2, to an SL receptor (Arellano-Saab et al. 2021). The W153L and F157T directly result in a larger more accessible pocket. The direct implication of the G189T mutation remains unclear, yet the combination of these mutations enabled the receptor to behave more promiscuously and perceive a larger diversity of SL ligands.

A selection of reported residues with functional significance for ligand selectivity are highlighted in Table 2. It is clear that many alterations in charge, size, and hydrophobicity will modify the binding-pocket morphology and selectivity, however, the precise mechanism(s) of how exactly these differential substitutions fine tune ligand specificity as well as the receptor’s catalytic function remain to be further explored both in vitro and *in planta*. In the following section we discuss the mode of action of D14 as SL hydrolases.

## Hydrolysis of strigolactones by D14 family hydrolases

### The catalytic triad and SL hydrolysis mechanism

**Fig. 5:**
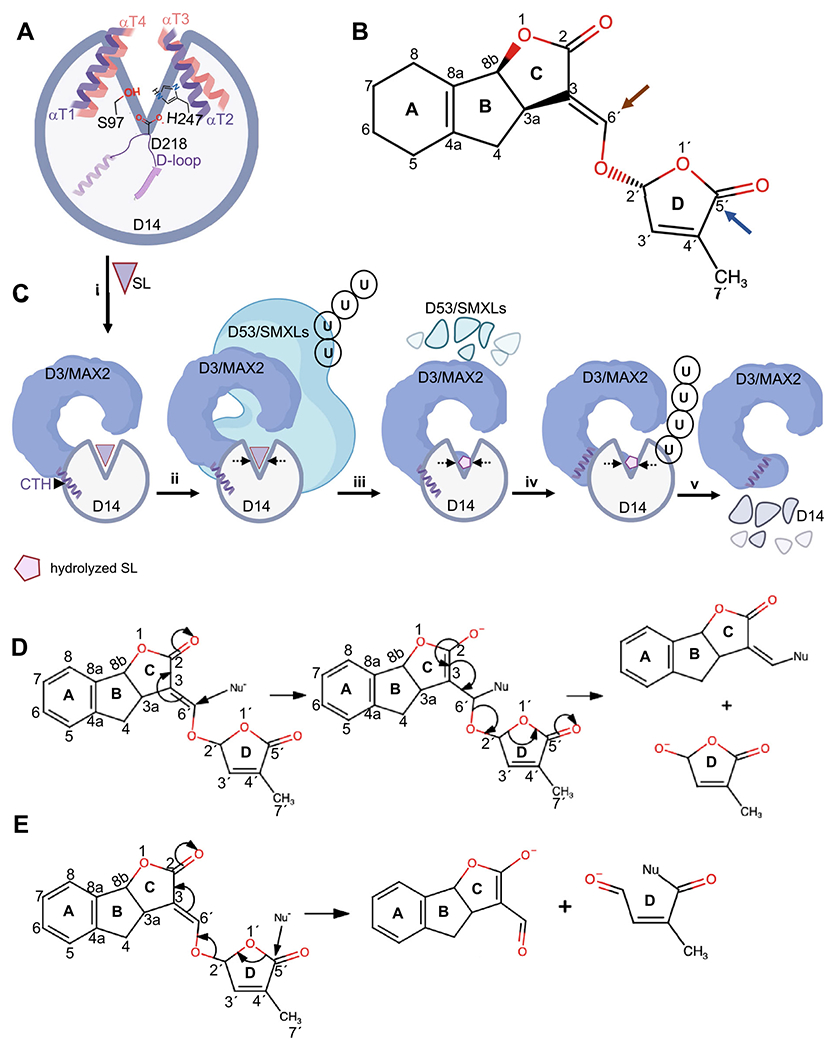
Proposed mechanism for strigolactone hydrolysis and signaling. **A** Schematic representation of SL receptor D14. V-shaped helices (αT1-αT4) are shown as cartoon in the open conformation and the catalytic triad residues serine, histidine, and aspartic acid (denoted S97-H247-D218) are shown in sticks. **B** Representative structure of SL (shown as ( +)-GR24). The brown and blue arrows indicate the proposed nucleophilic attacks on carbons 6′ or 5′. **C** Proposed model for strigolactone signaling pathway. ***(i)*** SL (purple triangle) is perceived by D14 (open conformation). D3/MAX2 with a dislodged C-terminal helix (CTH) recruits D14-SL and inhibits SL hydrolysis. ***(ii-iii)*** D3/MAX2-D14-SL targets and polyubiquitinates (U denotes ubiquitin) D53/SMXLs resulting in proteasomal degradation of D53/SMXLs, concurrent hydrolysis of SL (pink pentagon), and conformational changes in D14 (indicated by dashed arrows). ***(iv-v)*** Post SL hydrolysis, D14 is subsequently targeted by D3/MAX2 and degraded by the proteasome. Schematics were created with Bio-render.com **D, E** Proposed mechanisms of SL hydrolysis which results in D-ring and ABC-scaffold products by initiating nucleophilic attack on enol-ether linkage at C6′ **D**, or by initiating nucleophilic attack on C5′ **E**

In all D14s, the serine-histidine-aspartic acid catalytic triad is highly conserved and positioned at the bottom of the ligand-binding pocket (Fig. 4B and Fig. 5A) (Bythell-Douglas et al. 2017). D14s catalytic triad operates as a charge relay system together with an oxyanion hole that can stabilize high-energy transition states and hydrolysis intermediates of SL. Therefore, amino acid substitutions in the catalytic site result in a loss or decrease of SL-sensitivity (Hamiaux et al. 2012; Abe et al. 2014; de Saint Germain et al. 2016; Seto et al. 2019; de Saint Germain et al. 2021).

The mechanism of ligand hydrolysis by D14 has been a topic of interest and debate since before the formal identification of the receptor itself. The first proposed mechanism was generated by Mangnus and Zwanenburg in 1992 and was based on the activity of a unique synthetic SL on *Striga* germination. This study used a modified ‘reduced’ GR24 in which the enol-ether double bond is replaced by a single bond (Mangnus and Zwanenburg 1992). This modified GR24 failed to induce germination of *Striga* and *Orobanche* species and led to the suggestion that the enol-ether linkage connecting the C and D-ring is essential to retain activity. Hence, a reaction scheme was proposed in which the hydrolysis proceeds through a nucleophilic attack on enol-ether linkage (Fig. 5B and D) which results in two products: a D-ring (as a leaving group), and an intact ABC-scaffold (Mangnus and Zwanenburg 1992).

Later, Fukui et al. examined the effects of synthetic SLs, debranones (Fig. 3), on SL biosynthesis rice mutants. Debranones lack the enol-ether linkage can rescue the SL-biosynthetic mutant phenotypes and serve as bioactive SL, therefore providing counter evidence to the Magnus 1992 hypothesis (Fukui et al. 2011). Similarly, at the same year, Zwanenburg and Mwakaboko synthesized yet additional SL mimics, one of which also lacked the enol-ether linkage and was highly bioactive in stimulating *Striga* and *Orobanche* germination despite lacking this previously expected crucial linkage (Zwanenburg and Mwakaboko 2011).

After the discovery of the SL receptor, D14, and the identification of the homologous signaling pathway of KARs, a new model was proposed that could explain how both ligands can be hydrolyzed independent of the enol-ether bridge (Scaffidi et al. 2012). This mechanism follows a Michael reaction wherein a nucleophilic attack by the catalytic serine at the C5′ position of the D-ring results in similar products (D-ring and ABC scaffold) (Scaffidi et al. 2012) (see Fig. 5B and E for more details). This hydrolysis mechanism has been widely accepted as the most probable mode of action and is further supported by biochemical and structural evidence (Zhao et al. 2013; Nakamura et al. 2013; Yao et al. 2016; Takeuchi et al. 2018; Yao et al. 2017; de Saint Germain et al. 2016; Guercio et al. 2022).

To corroborate the proposed reaction mechanisms for SL hydrolysis, methods in structural biology and Mass Spectrometry (MS) have been used to determine various intermediate states in ligand hydrolysis. Two studies by Yao et al. have proposed an intermediate state where the D-ring of SL becomes covalently linked between the catalytic serine (at C5′ of SL) and the histidine (at C1′ of SL) (Fig. 5A, B, C and E) (Yao et al. 2016, 2017). Another study identified a stable hydrolysis intermediate where the D ring is covalently linked to the catalytic histidine of the receptor (de Saint Germain et al. 2016). This study further showed that the conserved methyl group at the 7′ position of SL is required for the reaction mechanism. Here, after the first nucleophilic attack by serine on C5′ of SL, there is an additional nucleophilic attack by the nitrogen atom of the histidine’s imidazole on the aldehyde intermediate at the C2′ of SL thus forming a covalent bond with the D ring (Fig. 5A, B, C and E) (de Saint Germain et al. 2016). A recent study on the crystal structure of pea KAI2 reported yet another form of SL intermediate covalently linked to the catalytic serine. This study also found the previously identified SL-histidine intermediate using MS on pea KAI2 (Guercio et al. 2022). Taken together, the favored mechanism of SL hydrolysis requires a successful nucleophilic attack on the C5′ of SL by the catalytic serine and is likely followed by multiple intermediate states. These covalently linked intermediates have been proposed to alter the receptors enzymatic activity and supported by the relatively slow turnover rates of D14s (Hamiaux et al. 2012; de Saint Germain et al. 2016; Shabek et al. 2018; Guercio et al. 2022; Tal et al. 2022). Despite the advancement in understanding the enzymatic aspects of SL receptors, the precise functional ramification of SL intermediate states on the perception and/or downstream signaling events remains to be further elucidated.

## Impacts on perception and hydrolysis of strigolactone by signaling partners

Perception and hydrolysis of SLs has been considered to be the very first step of the much larger cascade of the signaling pathway. Three key proteins are required to transduce the SL signal: the SL receptor D14, the D3/MAX2 (DWARF 3/ MORE AUXILLARY BRANCHES 2) ubiquitin (Ub) ligase F-box protein, and the target for proteasomal degradation, the transcriptional co-repressor proteins D53/SMXLs (DWARF 53 / SUPRESSOR OF MAX2-LIKE) (Nelson et al. 2011; Zhou et al. 2013; Soundappan et al. 2015; Wang et al. 2015; Kerr et al. 2021; Tal et al. 2022). These key players have been shown to form an SL-dependent protein complex and facilitate the ubiquitination and degradation of D53/SMXLs (Zhou et al. 2013; Shabek et al. 2018; Tal et al. 2022) (Fig. 5A and C). To date, the chronology of the binding events, and whether SL hydrolysis is required for formation of the signaling complexes, remains to be resolved.

Through structural examinations in vitro, one model has been proposed wherein SL hydrolysis is required for complex formation (Yao et al. 2016). A crystal structure revealed an SL-hydrolysis state with an altered structure of D14 in a complex with D3. Here, instead of the wide-open V-shaped α-helices of the lid, D14 is found in a closed conformation forming an additional interface with the rice D3 (Fig. 5A and C). This model suggests that the induced conformational changes during hydrolysis play an important role in the recruitment of D14 by D3.

In another model of SL complex formation, hydrolysis is not required for signal transduction (Seto et al. 2019). This is showcased by aspartic acid catalytic mutant, AtD14^D218A^, that lacks enzymatic activity yet can complement the *atd14* mutant phenotype in an SL-dependent manner. In this model, the active signaling state of D14 is triggered upon intact SL binding, but not by hydrolysis intermediate(s) or product(s). Therefore, it is proposed that the hydrolysis functions to deactivate the bioactive SLs postsignal transduction, but not for complex formation. Given the slow turnover of SL receptors compared to the proteasomal degradation rate of the target substrates in SL signaling, hydrolysis of SLs may not be required for D14-SL-D3-D53 complex formation (Hamiaux et al. 2012; Nakamura et al. 2013; Zhao et al. 2013).

This has been further supported by other studies (Shabek et al. 2018; Seto et al. 2019) centered on the findings that a pre-hydrolysis state of D14-SL is required for the recruitment of both D3 and its target protein D53/SMXLs. It has been proposed that a dynamic conformational change in the C-terminal helix (CTH) of D3 facilitates the recruitment of SL-bound D14-D53/SMXLs where D3 further attenuates the SL hydrolysis rate to allow effective ubiquitination and degradation of the D53/SMXLs (Shabek et al. 2018; Sobecks et al. 2022) (Fig. 5A and C). An intriguing mode of action has recently demonstrated that a primary metabolite such as citrate or citrate-like molecules could effectively trigger D3-CTH conformation and may serve as a link between specific environmental conditions and D3-D14-SL activation (Tal et al. 2022).

## Implications and conclusions

A deeper understanding of SL biosynthesis, their catalysis, and the structure of their receptors has allowed for the development of targeted agonists and antagonists not only as research tools, but also for agricultural applications. Synthetic SLs have the potential to serve a more potent or specific function than natural SLs and could provide a precise control of SL-influenced processes without off-target effects (Boyer et al. 2012). Hence, synthetic SLs hold promise as agrichemicals used in crop production and/or crop management strategies. For example, the development of synthetic SLs can aid to circumvent the devastating effect of SL-induced parasitic plant germination. *Striga* infestation impacts Sub-Saharan Africa, the Middle East, and parts of Asia, particularly by parasitizing and decimating staple cereal crops such as maize and sorghum (Parker 2009; Jamil et al. 2021). To this end, various agonists were developed to stimulate germination of *Striga* without a host, which will result in death of the obligate parasite (Toh et al. 2014; Takahashi and Asami 2018). This provides a crop management strategy for cereal farmers to deplete the soil of *Striga* before planting crops. Another strategy for crop protection is the development of antagonists that can target the *Striga* SL receptors to inhibit SL-dependent seed germination (Mashita et al. 2016; Takahashi and Asami 2018).

In addition, synthetic SLs can be utilized for precision farming to improve crops. Due to SLs’ role in inhibiting branching, SL antagonists can be used as yield stimulants by increasing the number of productive branches or tillers in crops such as grains (Mashita et al. 2016; Takahashi and Asami 2018). Alternatively, SL agonists can be applied to reduce the number of non-productive branches, especially in single-flower ornamental plants. SLs were also shown to impact secondary growth, and as such, SL agonists could be beneficial in increasing tuber yield of root vegetable crops as well as in stabilizing grain crops at risk of lodging (Agusti et al. 2011; Pasare et al. 2013; Takahashi and Asami 2018). The application of SL analogs could also increase crop tolerances to abiotic stress such as drought and salinity (Ha et al. 2014). The function of SL-induced AM symbiosis is yet another potential for utilizing synthetic agonists, especially due to the additional positive impacts of these symbioses including increased stress resistance and tolerance (Pozo and Azcón-Aguilar 2007; Evelin et al. 2009).

Overall, our understanding of the SL signaling pathway in the past decade has been increasingly advanced. In this review we highlight the central aspects of SL diversity, perception, and hydrolysis, however, there are many other facets and open questions that remain to be addressed. These include further elucidation of diversified SL biosynthesis, the precise molecular mechanism of the signal transduction cascade, the implications of SL crosstalk with other phytohormones, and the application of SLs in agriculture.

## Figures and Tables

**Table 1:** Diversity in SL binding pocket structures. *Oryza sativa* OsD14 crystal structure (PDB ID: 3W04) is shown in cartoon (gray) as a representation for the pocket orientation (purple, header). Solvent accessible surface areas were generated to represent the SL-binding pocket shape for: OsD14; *Arabidopsis thaliana* (At) AtD14 (PDB ID: 4IH4); *Petunia hybrida* DAD2 (PDB ID: 4DNP); *Saccharum spontaneum* SsD14a (PDB ID: 7F5W); *Striga hermonthica* ShD14 (PDB ID: 6XFO), ShHTL1 (PDB ID: 5Z7W), ShHTL4 (PDB ID: 5Z7X), ShHTL5 (PDB ID: 5CBK), ShHTL7 (PDB ID: 5Z7Y), ShHTL8 (PDB ID: 6J2R); *Physcomitrella patens* PpKAI2C (PDB ID: 6ATX), PpKAI2E (PDB ID: 6AZB), PpKAI2H (PDB ID: 6AZD); AtKAI2 (PDB ID: 4JYP); *Pisum sativum* PsKAI2B (PDB ID: 7K2Z). Residues defining pocket surface and solvent accessible (SA) volume were identified using CASTp with a 1.2 Å probe radius (Tian et al. 2018). Structural illustrations, and pocket surface calculations were generated and analyzed using PyMOL Molecular Graphics System, Schrödinger, LLC *Oryza sativa* OsD14 crystal structure (PDB ID: 3W04) is shown in cartoon (gray) as a representation for the pocket orientation (purple, header). Solvent accessible surface areas were generated to represent the SL-binding pocket shape for: OsD14; *Arabidopsis thaliana* (At) AtD14 (PDB ID: 4IH4); *Petunia hybrida* DAD2 (PDB ID: 4DNP); *Saccharum spontaneum* SsD14a (PDB ID: 7F5W); *Striga hermonthica* ShD14 (PDB ID: 6XFO), ShHTL1 (PDB ID: 5Z7W), ShHTL4 (PDB ID: 5Z7X), ShHTL5 (PDB ID: 5CBK), ShHTL7 (PDB ID: 5Z7Y), ShHTL8 (PDB ID: 6J2R); *Physcomitrella patens* PpKAI2C (PDB ID: 6ATX), PpKAI2E (PDB ID: 6AZB), PpKAI2H (PDB ID: 6AZD); AtKAI2 (PDB ID: 4JYP); *Pisum sativum* PsKAI2B (PDB ID: 7K2Z). Residues defining pocket surface and solvent accessible (SA) volume were identified using CASTp with a 1.2Å probe radius (Tian et al. 2018). Structural illustrations and pocket surface calculations were generated and analyzed using PyMOL Molecular Graphics System, Schrödinger, LLC.

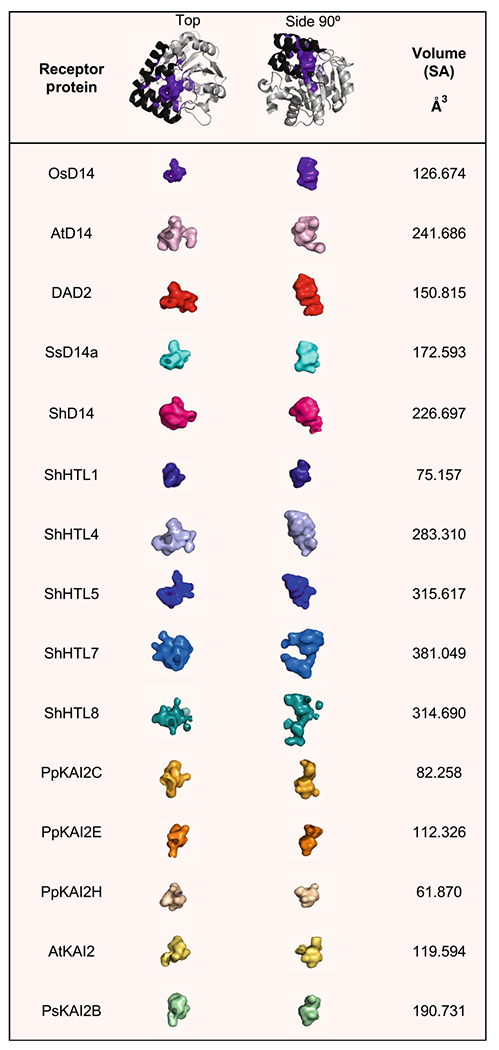

**Table 2:** Divergence in SL binding pocket residues. *Arabidopsis thaliana* D14 serves as the representative model. Column 1 shows amino acid position (based on PDB ID: 4IH4); column 2 shows the corresponding reported alternative residues in other plants. The equivalent position of each residue (green sphere) is shown in AtD14 (gray cartoon). The implications on the binding pocket as well as the research studies reporting the diverged residues are shown in columns 3 and 4. The 3D structure illustration and analysis were generated using PyMOL Molecular Graphics System, Schrödinger, LLC *Arabidopsis thaliana* serves as the representative model. Column 1 shows amino acid position (based on PDB ID: 4IH4); column 2 shows the corresponding reported alternative residues in other plants. The equivalent position of each residue (green sphere) is shown in AtD14 (gray cartoon). The implications on the binding pocket as well as the research studies reporting the diverged residues are shown in columns 3 and 4. The 3D structure illustration and analysis were generated using PyMOL Molecular Graphics System, Schrödinger, LLC.